# Emergence of a complex network structure on a Spatial Prisoner’s Dilemma

**DOI:** 10.1371/journal.pcbi.1013329

**Published:** 2025-08-12

**Authors:** Tomoko Sakiyama, Akihiro Takahara

**Affiliations:** 1 Department of Information Systems Science, Faculty of Science and Engineering, Soka University, Tokyo, Japan; 2 Information Systems Science, Graduate School of Science and Engineering, Soka University, Tokyo, Japan; NIT Rourkela EC: National Institute of Technology Rourkela Department of Electronics and Communication Engineering, INDIA

## Abstract

Many studies have proposed spatial game theory on network systems. Heterogeneous structures seem to contribute to population dynamics. However, few studies have addressed both dynamical population evolution and network growth events, especially incorporating individual players’ decision-making processes into the model. In this study, we considered a spatial prisoner’s dilemma (SPD) on a random network. In our model, the players were allowed to access the recent past information on themselves and neighboring players. In the “unlikely to happen” scenario, players adopted a strategy that rarely happens, which may have brought some risks to players. Moreover, the players in our model evolved their link with other players by altering their neighborhood when they received a low payoff. As a result, we found that our model spontaneously evolved as an approximate scale-free network around a critical parameter. Interestingly, hub players sometimes decreased their node degree; thus, these players are changeable in our system.

## Author summary

This study explores the coevolution of strategies and network structure in a spatial prisoner’s dilemma on a random network. Players adapt their strategies by occasionally adopting rare and risky behaviors that may not frequently occur but can influence outcomes. Moreover, when players receive low payoffs, they actively adjust their social links by changing neighbors, allowing the network to evolve dynamically. This interplay between strategy updates and network rewiring drives the spontaneous formation of an approximate scale-free network near a critical parameter value. Interestingly, players who become hubs in the network do not maintain constant connectivity; instead, their number of connections fluctuates over time. These results emphasize the importance of incorporating both individual decision-making processes and network evolution in models of population dynamics. Understanding such mechanisms provides valuable insights into how complex social and biological networks grow and change over time.

## Introduction

Spatial game theory demonstrates population evolutions in which both defective and cooperative players interact with each other [1–6]. Animals, including humans, sometimes behave selfishly, whereas at other times they behave cooperatively, depending on time and situation [7–9]. Thus, the dynamical co-evolutions of two strategies illustrate the complicated evolutions of populations, which are based on interactions among their agents.

Spatial game theories have been developed and applied in evolutionary population dynamics, with the spatial prisoner’s dilemma (SPD) model widely used as a representative framework [6,10,11]. Similar to other spatial game theories, a table, which is a payoff matrix among any two players in SPD populations, is used to calculate the earned payoffs for individual players. In SPD, a defector facing the opponent earns a benefit called Temptation (T), whereas the opponent cooperator obtains a Sucker (S) item. Two defectors obtain a Punishment (P) from each other. Conversely, two cooperators can earn a Reward (R) when they meet each other. Players who earn payoffs interact with one another, and they sometimes update their strategy with the opponent based on the earned payoffs, resulting in the development of individual strategies among populations. In addition, in classical game theories, including SPD, the cooperative populations are invaded by selfish populations within some range of payoff matrix parameters [11]. In SPD, cooperative populations almost disappear when defective players facing the opponent players earn payoffs of a certain extent, that is, in case of high temptation (T) values. Therefore, numerous spatial game theory models have been developed for the evolution of cooperative behaviors [12–19].

Spatial game theory can form a class of games from biology, social and physics modeling of the evolution; thus, it has been applied not only to the lattice environment, but also to the network environment [20–28]. Moreover, emergent cooperative behaviors in spatial game theory can be dependent on the system topology, and many studies have reported on the evolution of cooperative behaviors using spatial game theory on the network systems [25–30].

Social and biological networks in the real world exhibit the characteristic network property of heterogeneous structures [31–33]. For examples, real world networks present a scale-free property [34]. A scale-free property refers to a network’s degree distribution, which follows a power law. A power law distribution has no characteristic scale, and it gradually decreases with the increase of degrees. Several studies have focused on the application of spatial game theory to static heterogeneous network structures and revealed that a heterogeneous network structure may enhance the cooperative populations [20,25,27,28]. However, real-world network structures can be drastically changed; therefore, the network systems can grow, which may result in the emergence of a heterogeneous property [34–37]. In that context, several studies have examined how network growth processes—such as link rewiring—interact with game dynamics like the spatial prisoner’s dilemma (SPD). These studies have primarily focused on the promotion of cooperation and the emergence of heterogeneous network structures [38–43], including scale-free structures [44, 45]. However, relatively little attention has been paid to game-theoretic models that focus on how players make decisions about whom to connect with or disconnect from, based on their own payoffs or scores, during the dynamic formation of network structures [46].

Thus, in this study, a spatial game theory model was developed on a network system to address the co-evolution of cooperative behaviors and a heterogeneous network. Our model included two features regarding the strategy update and the link evolution. In the strategy update event, we considered the risk-taking aspect regarding the memory use of the players, because a risk-taking approach is an important aspect in decision-making processes [47 and 48]. Here, we allowed individual players to consider the recent past information on themselves and the neighborhoods. The memory use of the players has been widely studied in SPD [49–52]. However, many studies have focused on the long-term memory use, which might be related to the emergence of cooperation [49 and 50]. In those studies, the players were allowed to access to the long-term past information and they used the past information in their decision-making processes. Furthermore, mentioned above, humans sometimes take risks. Therefore, the players in our model rather attributed attention to the past information that was unlike to happen and actively attempted to use it [53].

Regarding the connection/disconnection link, the players in our model either were attracted to a newly encountered, higher-scoring player or chose to sever ties with it [35 and 42], based on their own and their partner’s scores or payoffs. Thus, the players in our model attempted to evolve their link to alter their surrounding situations. We found that our model exhibited dynamical population evolutions in which cooperators were able to survive, even in the case of high Temptation (T) values. Moreover, the system exhibited an approximate scale-free network structure, in which the hub players were sometimes changeable.

## Methods

### Space and agents

We set 1,000 nodes on a random network system. Here, each node was an individual agent (player). A relationship whether two players play the cooperation-defection game with each other was represented by a link between them. The initial strategies of the players were randomly assigned to individuals, i.e., their strategies were either defector or cooperator ones, with an equal probability. We started each simulation using a random graph as a network topology. The average degree of nodes at the beginning of the trials was set to 5. We ran each simulation trial for 100,000-time steps.

### Model description

We considered the weak version of the PD game, as follows: R=1, S=P=0, T=b, 1<b<2 [10]. The payoff matrix is shown in Table 1. The perverse prisoner’s dilemma (PPD) network growth model was composed of several sub-models [54]. We used a synchronous update.

**Table 1:** Payoff matrix.

	Defector	Cooperator
Defector	P (0)	T (*b*)
Cooperator	S (0)	R (1)

### Process overview

1. On time *t* iteration:
2. Sub-model: *Score calculation*.

Individual players interact with their adjacent players and earn their score using a payoff matrix.

1. Sub-model: *Strategy update*.

In this event, individual players update their strategy using the SPD classical update rule or the memory-based rule. They choose one of these two update rules based on their circumstances. In the SPD classical update rule, individual players compare their calculated scores with those of their adjacent players and update their strategy with one of their adjacent players who earns the highest score. In the memory-based rule, players estimate their next strategy based on the recent frequency of each strategy.

1. Sub-model: *Network growth*.

Individual players sometimes connect/disconnect to/from a player. At one time point, players likely connect to an unknown player who is a friend of their friend (link connection). At another time point, players likely stop being friends with one of their friends (link disconnection).

1. Time *t* is changed to *t*+1.

### Submodels

In this subsection, we describe the different components of our model in detail. Although all players are processed synchronously in a similar way in each sub-model, we focus on player i as an example.

1. Score calculation

In this sub-model, player i interacted with all of its adjacent players and earned its score (${score}_{i}$ indicates the score for player i using the payoff matrix). Scores were calculated by playing against adjacent players, with each score being computed and then accumulated.

1. Strategy update

After calculating the score, player i updated its strategy using one of the two different strategy update rules. If the score for player $i\;({score}_{i}$) is equal to the highest score among itself and its adjacent players, then it uses the classical SPD rule. In the classical SPD rule, player i compares ${score}_{i}$ with those of their adjacent players and update its strategy (${strategy}_{i}$) with one of their adjacent players who earns the highest score.

In contrast, if the score of player $i\;({score}_{i}$) is smaller than the highest score among itself and its adjacent players, then it uses the memory-based update rule. In this case, player i counted the number of cooperators among itself and its adjacent players at the current time (*t*) and at one previous time point: ${count}_{i}^{t}$ and ${count}_{i}^{t-1}$. Here, a variable ${count}_{i}^{t}\;$indicates the number of cooperators among itself and i*t*s adjacent players at time *t* for player i [54]. Since past information is not available at time 0, the classical SPD rule is used.

Two update rules are in accordance with the following expression:

$if\;t=0\;or\;i\in {MAX\omega }_{i}^{t}(t>0),$

$if\;{strategy}_{j}={strategy}_{k}\;for\;all\;j\in {MAX\omega }_{i}^{t}\;and\;all\;k\in {MAX\omega }_{i}^{t}\;(j\neq k),$


$${strategy}_{i}\;={strategy}_{j}\;$$ (1)

if not,


$${strategy}_{i}\;={strategy}_{i}$$ (2)

where $\omega_{i}^{t}$ indicates the set of itself and adjacent players for player i at time t, whereas ${MAX\omega }_{i}^{t}$ indicates the set of itself and adjacent players for player i at time t who have the highest score within $\omega_{i}^{t}$.

$if\;i\notin {MAX\omega }_{i}^{t}(t>0),$

With a probability $\frac{{count}_{i}^{t}\;+\;{count}_{i}^{t-1}}{N_{i}^{t}\;+\;N_{i}^{t-1}},$


$${strategy}_{i}=defector$$ (3)

whereas with a probability $1-\frac{{count}_{i}^{t}+{count}_{i}^{t-1}}{N_{i}^{t}+N_{i}^{t-1}}$,


$${strategy}_{i}=cooperator\;$$ (4)

where $N_{i}^{t}$ represents the total number of players, including itself and adjacent members, for player i at time *t*, which satisfies the following equation:


$$N_{i}^{t}=\left|\omega_{i}^{t}\right|\;$$ (5)

3 Network growth

In our model, player i connected/disconnected to/from one player whenever it earned the lowest score among its adjacent players. The link connection/disconnection is controlled by a parameter p as follows.

$if\;i\in {MIN\omega }_{i}^{t}\;and\;\omega_{i}^{t}\neq \left\{i\right\},$

with a probability p,

player i  randomly selected a player j from its adjacent players. Subsequently, it connected its link with a randomly selected player k, who was an adjacent player of player j, but not of player i, and who had the highest score among j and j’s adjacent players, in accordance with the following equation:


$$k\in {MAX\omega }_{j}^{t}\;and\;k\notin \omega_{i}^{t}\;$$ (6)

with a probability (1−p),

where player i disconnected its link from a randomly selected player j who had the highest score among player i and its adjacent players, in accordance with the following equation:


$$j\in {MAX\omega }_{i}^{t}$$ (7)

where ${MIN\omega }_{i}^{t}$ indicates the set of itself and adjacent players for player i at time t who had the lowest score within $\omega_{i}^{t}$.

$if\;\omega_{i}^{t}=\left\{i\right\},$

player i connected its link with a randomly selected player j.

Therefore, at one time point, players were likely to connect to an unknown player who was a friend of their friend. At another time point, it was likely to stop being friends with one of their friends. A distinctive feature of the model was that an individual modified its links when it had the lowest score within its community, where the community was defined as the individual itself and its neighboring players. In such cases, with probability p, the individual attempted to improve its position by forming a link with the highest-scoring player who was connected to one of its current neighbors. Alternatively, with probability1−p, it severed its connection with its own highest-scoring friend.

## Results

**Fig 1:**
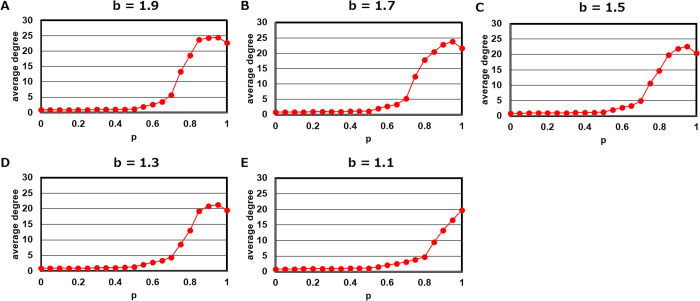
Average degree after each trial among over 100 trials for parameter p. **A.** b=1.9. **B.** b=1.7. **C.** b=1.5. **D.** b=1.3. **E.** b=1.1.

First, we examined the relationship between the parameter p and the link evolution using several b values (b=1.1, 1.3, 1.5, 1.7 and 1.9). Here, we obtained the average degree of nodes at the end of the simulations from 100 trials. According to Fig 1, the system exhibited a phase transition around p=0.75.

**Fig 2:**
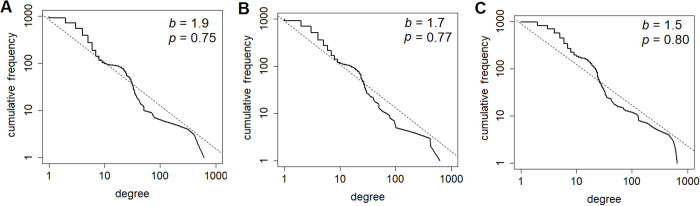
Relationship between the node degree and its cumulative frequency after one trial. **(A)** b=1.9, p=0.75. **(B)** b=1.7, p=0.77. **(C)** b=1.5, p=0.80. The dashed lines indicate the best fit power-laws.

Several previous studies have focused on high b values because defectors can only become winners against the opponent players [53]. We investigated the analysis regarding network structures using several high b values (b=1.5, 1.7 and1.9). we examined the properties of node degrees because real-world networks sometimes present complex characteristics such as scale-free networks. According to Fig 2, the system exhibited an approximately scale-free structure characterized by humps (Fig 2A (b=1.9, p=0.75): γ=1.71, AIC weights for a power-law against an exponential-law = 1.00, Fig 2B (b=1.7, p=0.77): γ=1.71, AIC weights for a power-law against an exponential-law = 1.00, Fig 2C (b=1.5, p=0.80): γ=1.58, AIC weights for a power-law against an exponential-law = 1.00). Here, node degrees were obtained for individual players at the end of one trial. The parameter p for individual b values were fine-tuned around p=0.75 to obtain an approximate power-law. The fraction of nodes with degree k in the scale-free network was according to$\;P(ksimk^{-\gamma }$. These results suggest that a scale-free structure emerged to some extent within a certain range of the parameter b.

To verify the system dynamics in greater detail, we set b=1.9 and p=0.75. The population dynamics are reported in S1 Fig as a time-dependent analysis with, suggesting that both strategies were well-mixed until the end of the calculation. Thus, our model does not allow defective population to be dominant even at high temptation (T) values.

**Fig 3:**
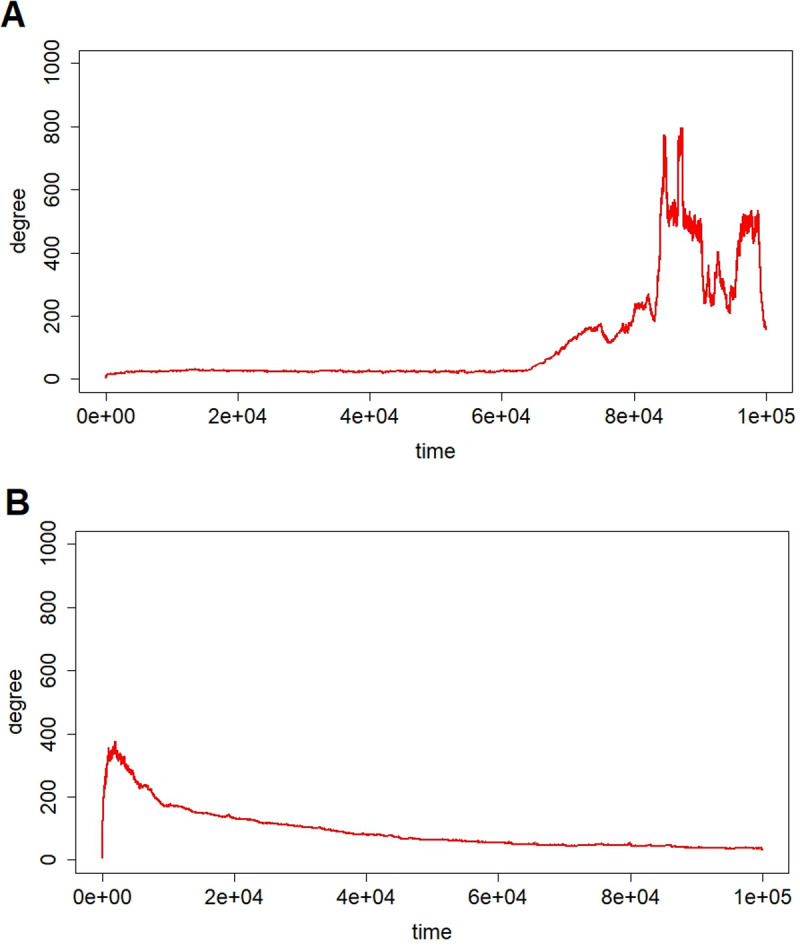
Examples of the node evolutions of two different players using b=1.9 and p=0.75.

Fig 3 provides several examples of time evolutions for node degree. Here, the time evolutions for node degree of two players are depicted as examples. It was evident that both players became hub nodes over time. Interestingly, one of the players in Fig 3A retained the role of a hub node, whereas the other player in Fig 3B decreased its node degree subsequently, suggesting that hub nodes do not always remain hub nodes.

**Fig 4:**
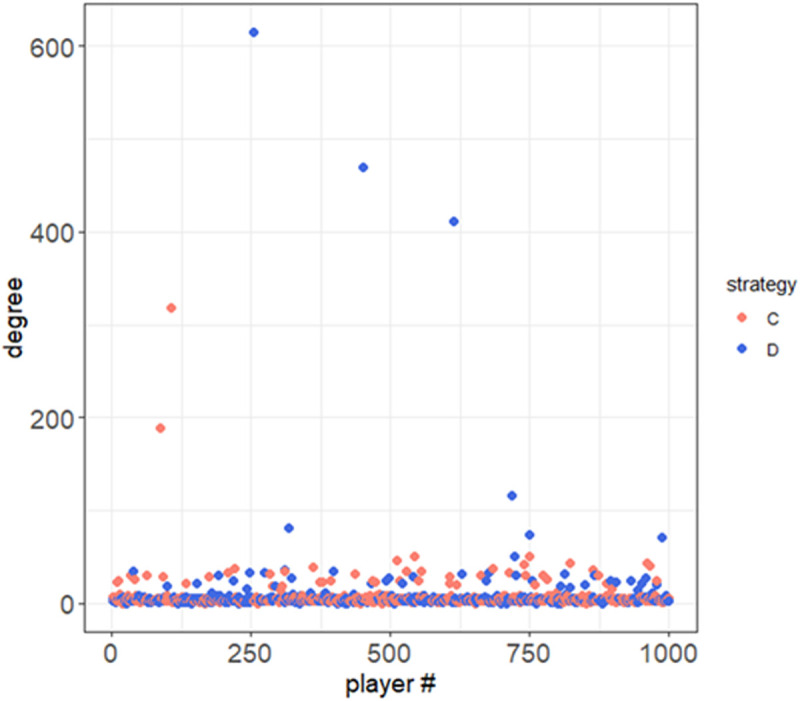
Relationship between the player # and their node degree at the end of trial for each strategy using b=1.9 and p=0.75.

Next, to evaluate the relationship between the strategies and node degree of the players, we calculated that relationship at the end of the trial. We focused on populations including more than 100 node degrees according to time evolution, and found that the ratio of defectors vs. cooperators in those populations was 0.73 ± 0.036, as derived from 100 trials. In fact, Fig 4 presents a one-trial example of the relationship between node degrees and strategies for players at the end of the trial, suggesting that both strategies can be hub nodes, whereas defectors are likely to become hub nodes. This may be explained by the possibility that the players are likely to establish a new link with a defector, because defectors can earn higher scores against the opponents.

**Fig 5:**
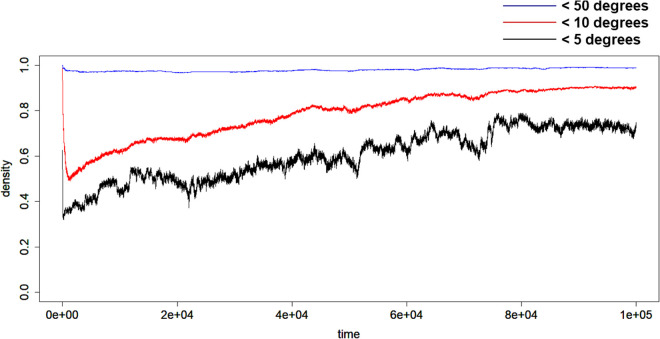
Fraction of players having less than certain node degrees using b=1.9 and p=0.75: less than 5 degrees (<5 degrees), less than 10 degrees (<10 degrees), and less than 50 degrees (<50 degrees).

As shown in Fig 5, which provides an example of the fraction of players’ nodes that satisfied less than certain degrees, almost all players had small node degrees, whereas a few players were hub nodes. Interestingly, this tendency was not dependent on the time evolution after a certain time threshold. While the system consistently contains hub nodes, as more clearly illustrated in Fig 3, it is also evident that the player serving as the hub may change dynamically over time.

We also examined the relationship between the parameter b (temptation to defect) and the evolution of cooperation, based on the results of 100 simulation trials (S2 Fig). In this analysis, we tested three different values of the parameter p: 0.75, 0.55 and 0.25. First, there is a clear trend that a smaller value of parameter p leads to an increase in the number of defectors. This tendency is likely related to the decrease in the average node degree as p decreases (Fig 1). Indeed, as shown in S3 Fig, as the parameter p decreases, the system includes a greater proportion of cases in which cooperators are less likely to be adjacent to one another. Notably, although the average proportion of cooperator adjacency at p=0.55 is still higher than at p=0.75. S3 Fig was generated using b=1.9 and for each value of p, it shows the proportion of neighbors that are cooperators for each cooperative player at the end of a single simulation trial. For p=0.55 and p=0.25 in S2 Fig, the density of defectors shows little change as the parameter b becomes larger. However, when p=0.75 the density of defectors tends to slightly decrease as b increases. This can be explained by the fact that, as shown in Fig 1, a higher value of p is required to trigger a phase transition when b is small.

To examine the network dynamics in greater detail, we calculated the degree correlation function $k_{nn}$, defined as the average degree of the neighbors of a player with degree k. S4A Fig presents a representative example from a single trial with parameters b=1.9 and p=0.75, suggesting that hub nodes (i.e., players with high degree) are not directly connected to one another. Indeed, highly connected players, whether defectors or cooperators, tend to form connections with both defectors and cooperators, indicating that hub players are not inclined to preferentially connect with others who share their strategy (S4B and S4C Fig). Here, S4B and S4C Fig show the average proportion of neighboring players who are cooperators and defectors, respectively, for cooperative and defective players, categorized by node degree at the end of a single trial (b=1.9 and p=0.75). This result is consistent with the observation shown in S3 Fig, which indicates that when the parameter p takes a large value (e.g., p=0.75), the proportion of cases in which cooperators are adjacent to each other becomes higher.

Next, we checked the parameter effects. Here, we replaced the initial average degree for individual players, i.e., 5.0, with 2.5 or 10.0, and found that the system yielded similar results with the default parameter regarding the average degree of nodes at the end of the trials (100 trials) (S5 Fig). Several studies have reported that the method of score calculation can influence the outcomes of the system [25 and 55]. Therefore, we also examined the effects of score calculation using an average-based framework. In this framework, individual accumulated scores are averaged by the degree of each player. As shown in S6A Fig, which illustrates the relationship between the parameter p and the average node degree at the end of the trials with b=1.9, the system exhibits a phase transition at a smaller p value compared to the default accumulated scoring scenario. This may be due to the fact that scores tend to be smaller in the average framework, making players more likely to connect with others they prefer, based on the sub-model of network growth. Notably, the proportion of cooperators does not decrease under this framework (S6B Fig shows examples of time evolutions regarding the defector density).

Finally, we evaluated the importance of the memory use in our model. Here, we set the non-memory PPD model where players always used the classical SPD strategy update rule. The other procedures in the non-memory PPD model remained the same as those used in the PPD network growth model. We found that the almost all of the players become defectors (the last defector density = 0.99 ± 0.010 obtained from 100 trials). S7 Fig presents an example of the evolution of defector density. In this study, we set p=0.75. These results indicate that the non-memory PPD model cannot produce neither well-mixed populations nor any population fluctuations. These results confirmed that the memory use of players affords drastic population dynamics and network growths to the system.

## Discussion

Here, we developed a new SPD model on a network system. In the proposed model, the players considered it unlikely to happen regarding a strategy update and at times adopted an unlikely strategy based on the local circumstance. Furthermore, the players sometimes connected with a new player who was a friend of their neighbors or disconnected from a neighboring player by incorporating individual players’ decision-making processes into the model. By switching between these two events with appropriate probability for individuals, the system exhibited the emergence of a heterogeneous network: an approximate scale-free network with well-mixed populations, even though it was initially a random network. Defectors tended to become hub players, whereas cooperators also had the opportunity to become hub players. Occasionally, they decreased their degrees and were no longer hub players.

The memory use of players in spatial game theories has been widely studied because real organisms have some type of memory capacity [49–52]. It seems that players necessitate some ingenuity to produce cooperative behaviors when they are allowed to access to short-term memory [53 and 56]. In our model, the players considered an “unlikely to happen” scenario regarding the strategy update [53]. Importantly, such an unlikely to happen condition appeared to promote population evolutions and network growths using only limited memory capacities among the players. In that sense, the players do not necessarily need to have a long-term memory capacity.

In heterogeneous networks, only a few players can accumulate benefits, whereas many other players receive a low payoff [27]. Therefore, it may be a natural condition for players to connect with a new player who earns a high score. At the same time however, our model incorporates a mechanism in which the lowest-scoring player in a neighboring community may, with a certain probability, sever a connection with a higher-scoring player in order to escape their disadvantaged position. It appears that the replacement of the connection/disconnection rule with a specific probability (parameter p) enabled the system to enter a phase transition. Around a critical p value, the system exhibited an approximate scale-free network structure. Thus, link evolutions, including both connections and disconnections, are necessary for the formation of heterogeneous structures in a network system [46].

In summary, first, the players in our model were randomly connected with each other. However, as time elapsed, a heterogeneous structure appeared spontaneously. Some players became hub players. Furthermore, our model produces a well-mixed population even at high temptation (T) values. The decision-making processes for players of the characteristic network evolution in our model were as follows:1) a short-term memory use that focused on an “unlikely to happen” condition and 2) a link connection/disconnection in which the players changed their neighborhood situations. Our model can describe the game theory application in the socio-economic analysis since game theory impacts agents’ decision-making by offering a way to analyze how choices made by different agents influence one another. It highlights how the results depend on the strategies of all parties involved, focusing on their interdependent interactions. Moreover, our model can reveal the evolution of complex networks, which may provide comprehensive understanding of complex systems and evolutionary game theories.

## Supporting information

S1 FigExample of the time evolution for defector density using b=1.9 and p=0.75.The small square attached to the top of the figure represents an entangled view of the last 5,000-time steps.(TIF)

S2 FigDefector density after each trial among over 100 trials for parameter b using p= 0.75, 0.55 and 0.25.(TIF)

S3 FigThe proportion of neighboring cooperators for individual cooperative players after one trial using b=1.9 for three different p: 0.75, 0.55 and 0.25.(TIF)

S4 FigThe degree correlation function (A) and the proportion of neighboring cooperators/defectors for individual cooperative/defective players after one trial (B and C).Here, the parameters b and p were set to 1.9 and 0.75, respectively.(TIF)

S5 FigRelationship between the average node degree at the end of the trials and the parameter p obtained from 100 trials.A. The initial average degree for players was 2.5. B. The initial average degree for players was 10.(TIF)

S6 FigResults for the average framework in the scoring scenario.A. Relationship between the average node degree at the end of the trials and the parameter p obtained from 100 trials. B. Examples of defector evolution for two different values of p: 0.75 and 0.25. Here, the parameter b was set to 1.9.(TIF)

S7 FigExample of the evolution of the defector density at p=0.75 for the non-memory PPD model.(TIF)

S1 Data Dataset. (XLSX)

S1 Text Source code. (DOCX)
