# Supplementary figures and images for: Emergence of a complex network structure on a Spatial Prisoner’s Dilemma
Source: PLoS Comput Biol. 2025 Aug 12;21(8):e1013329. doi: 10.1371/journal.pcbi.1013329 (PMC12360660; doi:10.1371/journal.pcbi.1013329)

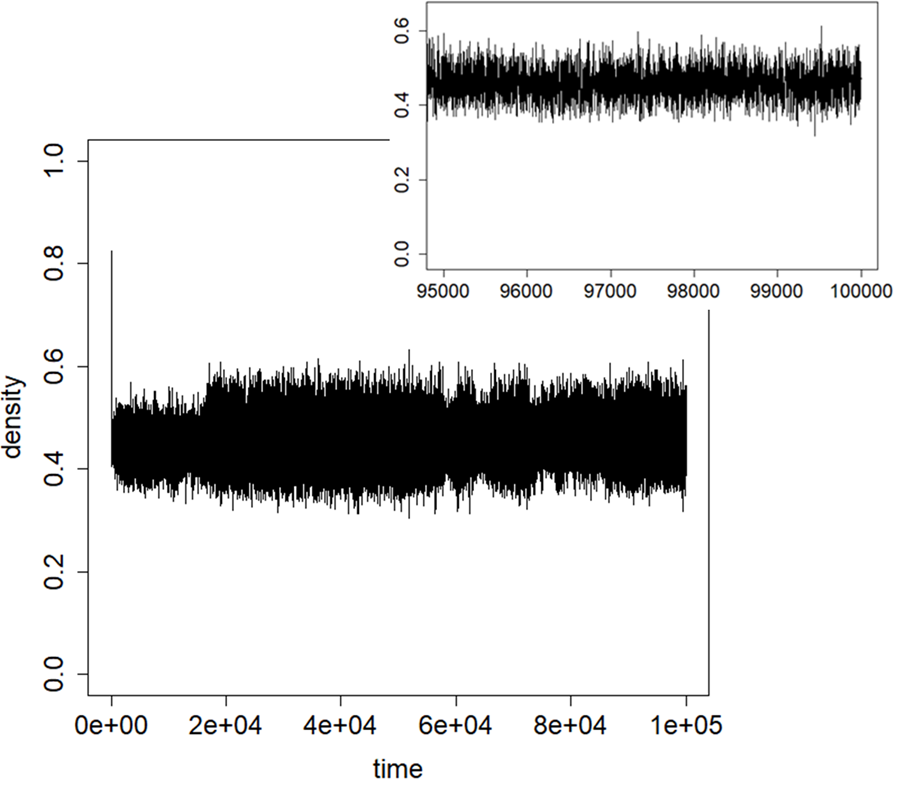

Supplement: S1 Fig — The small square attached to the top of the figure represents an entangled view of the last 5,000-time steps. (TIF) [file pcbi.1013329.s001.tif]

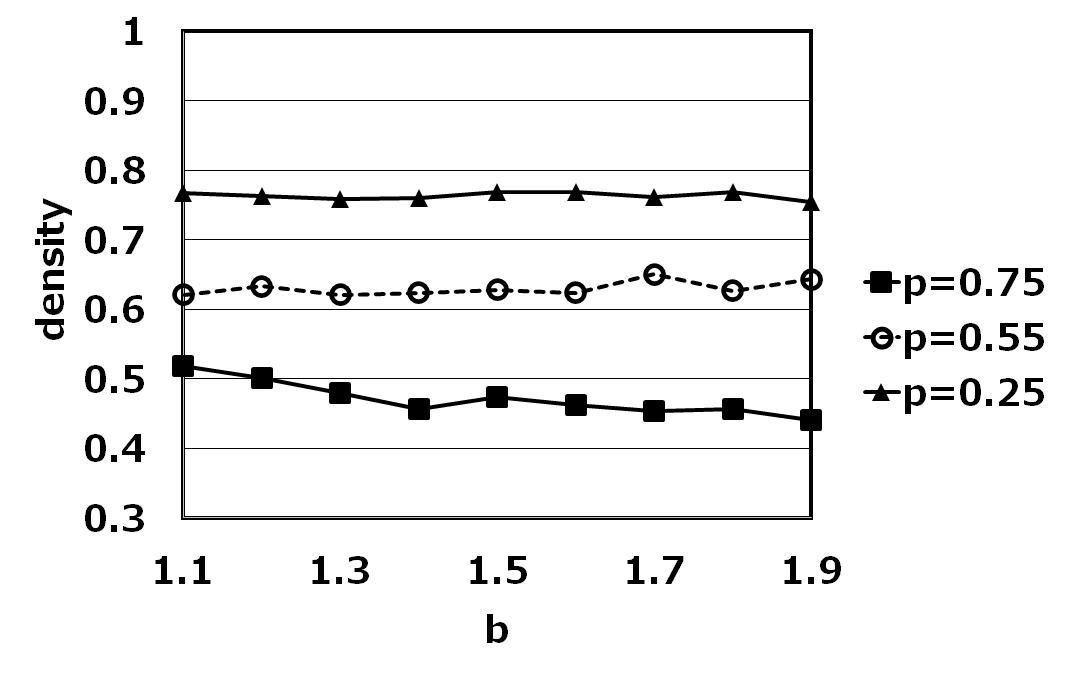

Supplement: S2 Fig — (TIF) [file pcbi.1013329.s002.tif]

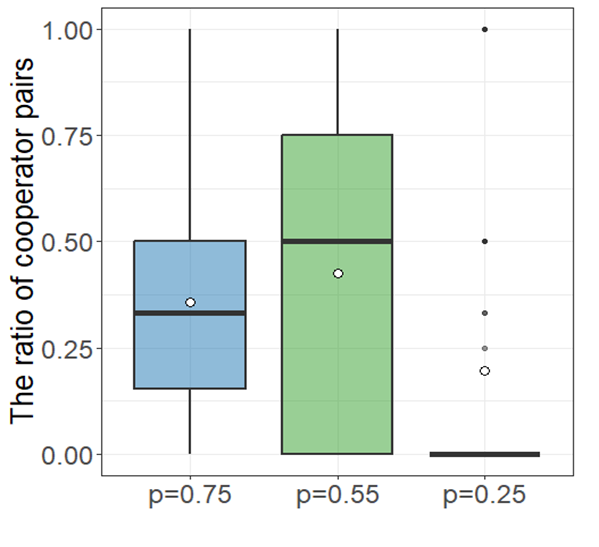

Supplement: S3 Fig — (TIF) [file pcbi.1013329.s003.tif]

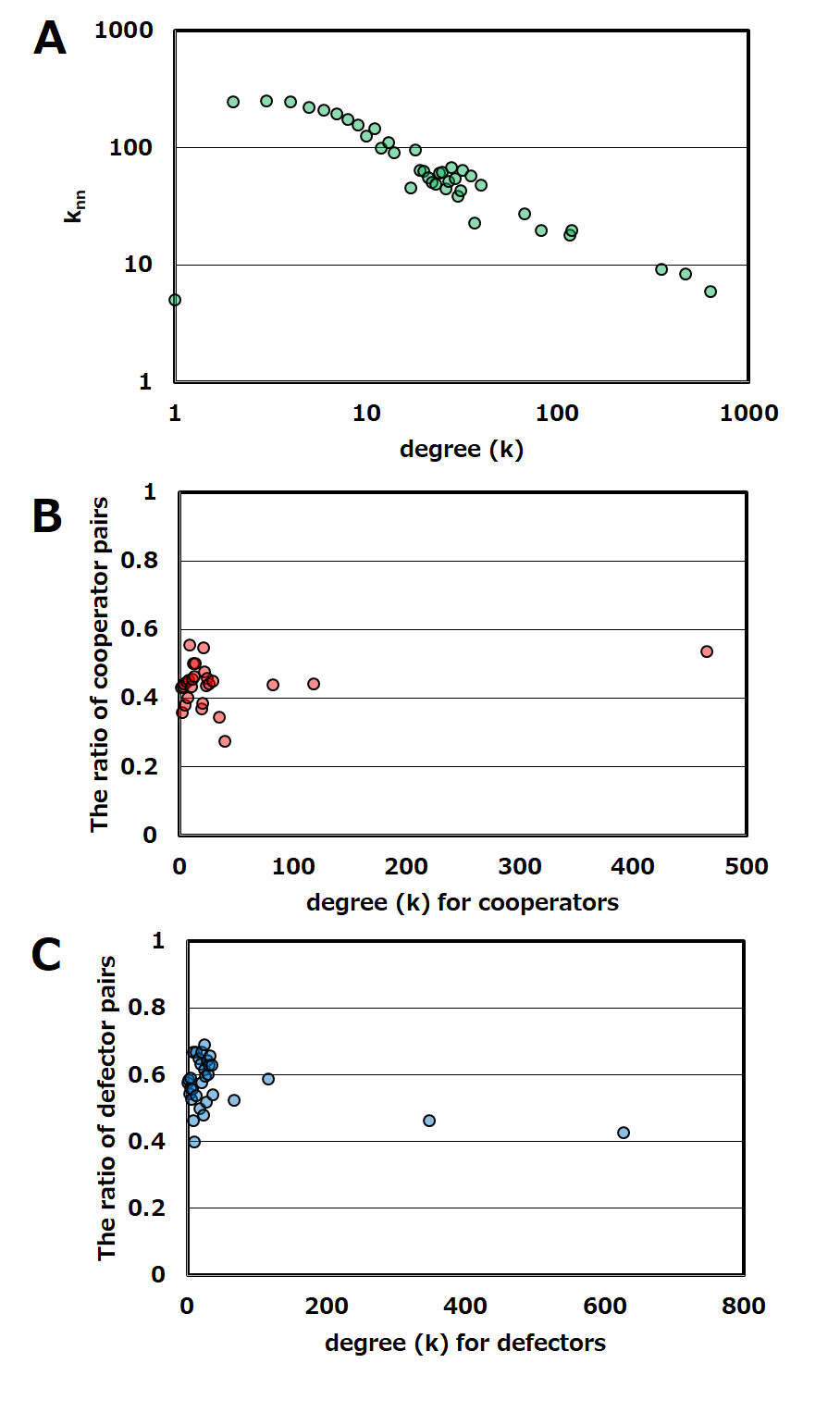

Supplement: S4 Fig — Here, the parameters b and p were set to 1.9 and 0.75, respectively. (TIF) [file pcbi.1013329.s004.tif]

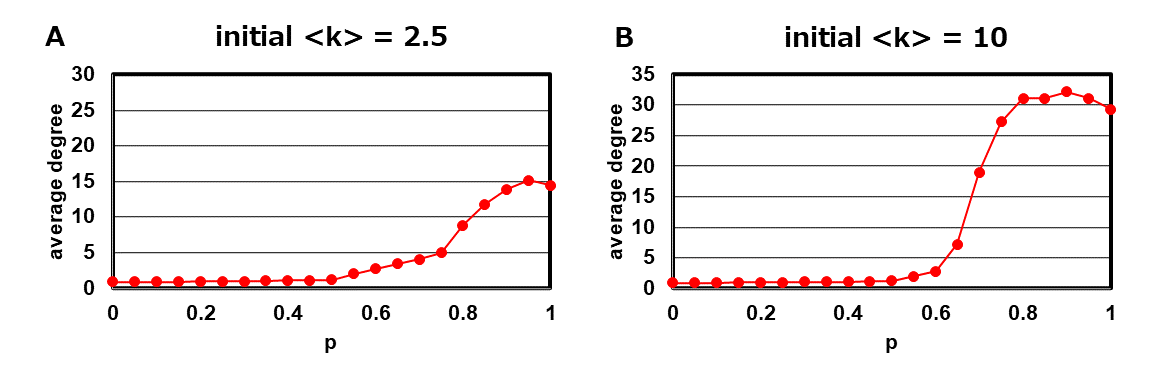

Supplement: S5 Fig — A. The initial average degree for players was 2.5. B. The initial average degree for players was 10. (TIF) [file pcbi.1013329.s005.tif]

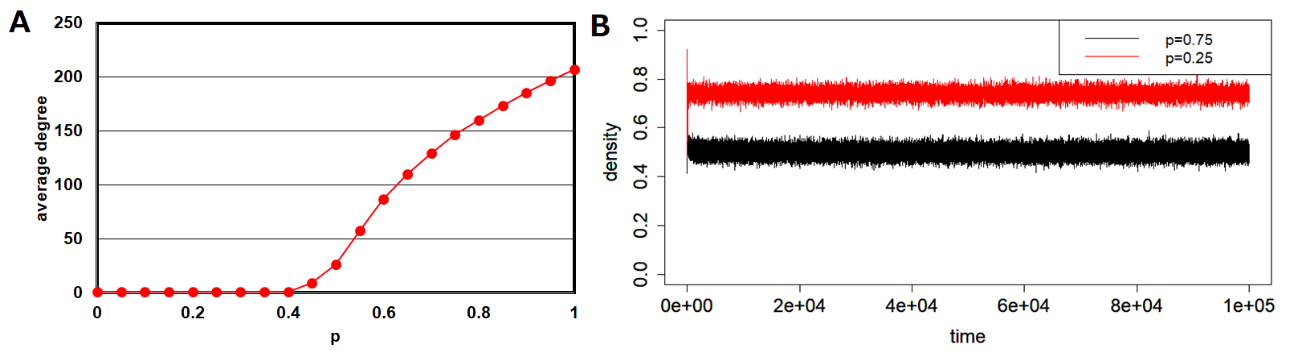

Supplement: S6 Fig — A. Relationship between the average node degree at the end of the trials and the parameter p obtained from 100 trials. B. Examples of defector evolution for two different values of p: 0.75 and 0.25. Here, the parameter b was set to 1.9. (TIF) [file pcbi.1013329.s006.tif]

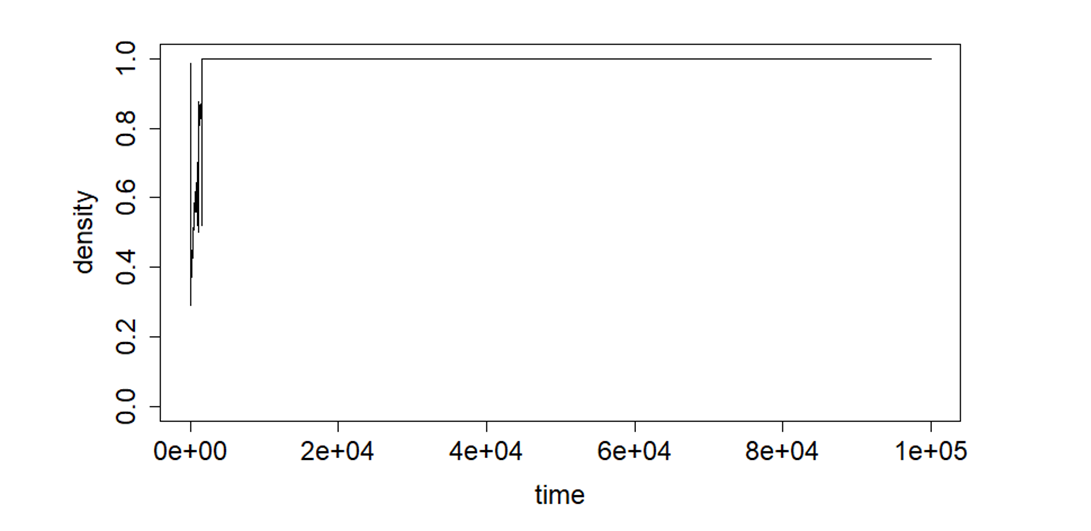

Supplement: S7 Fig — (TIF) [file pcbi.1013329.s007.tif]
