# Supplementary material for: Emergence of a complex network structure on a Spatial Prisoner’s Dilemma
Source: PLoS Comput Biol. 2025 Aug 12;21(8):e1013329. doi: 10.1371/journal.pcbi.1013329 (PMC12360660; doi:10.1371/journal.pcbi.1013329)
Supplement: S1 Text — (DOCX) [file pcbi.1013329.s009.docx]

/////////// Score calculation ////////////

//--------------------------------------------------------------------------------------------------------------

// Here, s[i] and paststrategy[t][i] represent the current strategy and the strategy at time t for player i.

// The variable score [i] means the current score for player i.

// The variable edge[i][j] indicates whether player i establishes a link with player j (1) or not (0).

//--------------------------------------------------------------------------------------------------------------

for(i=0;i<m;i++)

{

paststrategy[t][i] = s[i];

}

for(i=0;i<m;i++)

{

score[i] = 0;

for(j=0;j<m;j++)

{

if(edge[i][j] == 1)

{

if(s[i] == 0 && s[j] == 0)

{

score[i] += R;

}

else if(s[i] == 0 && s[j] == 1)

{

score[i] += S;

}

else if(s[i] == 1 && s[j] == 0)

{

score[i] += b;

}

else if(s[i] == 1 && s[j] == 1)

{

score[i] += P;

}

}

}

}

/////////// The strategy at the next time step is computed based on memory ////////////

//--------------------------------------------------------------------------------------------------------------

// The variable count[i] represents the number of players — including player i and its neighbors — whose strategy is Cooperator at the current time and the previous time step.

// The variable s_memory[i] represents the strategy at the next time step computed based on memory.

// The variable rn is a uniformly distributed random number satisfying 0<rn<1.

//--------------------------------------------------------------------------------------------------------------

if(0 < t)

{

for(i=0;i<m;i++)

{

count[i] = 0;

sum[i] = 0;

for(k=0;k<2;k++)

{

if(paststrategy[t - k][i] == 0)

{

count[i]++;

}

sum[i]++;

for(j=0;j<m;j++)

{

if(edge[i][j] == 1)

{

if(paststrategy[t - k][j] == 0)

{

count[i]++;

}

sum[i]++;

}

}

}

fraction[i] = (double)count[i]/(double)sum[i];

if(rn < fraction[i])

{

s_memory[i] = 1;

}

else

{

s_memory[i] = 0;

}

}

}

/////////// The strategy at the next time step is computed based on the classical SPD rule ////////////

//--------------------------------------------------------------------------------------------------------------

// The variable max_score[i] represents the highest score among player i and its neighbors.

// The variable top[i] is 1 if player i has the highest score among itself and its neighbors; otherwise, 0.

// The variable s_spd[i] represents the strategy at the next time step computed based the classical SPD rule.

//--------------------------------------------------------------------------------------------------------------

for(i=0;i<m;i++)

{

top[i] = 0;

max_score[i] = score[i];

max_strategy = s[i];

for(j=0;j<m;j++)

{

if(edge[i][j] == 1)

{

if(max_score[i] < score[j])

{

max_score[i] = score[j];

max_strategy = s[j];

}

}

}

y[i] = max_strategy;

if(max_score[i] == score[i])

{

top[i] = 1;

}

s_spd[i] = y[i];

// If two or more of the top-ranked players among player i and its neighbors have different strategies, then s_spd[i] is reset to s[i].

}

/////////// The strategy update ////////////

for(i=0;i<m;i++)

{

if(0 < t)

{

if(top[i] != 1)

{

s[i] = s_memory[i];

}

else

{

s[i] = s_spd[i];

}

}

else

{

s[i] = s_spd[i];

}

}

/////////// The network growth ////////////

//--------------------------------------------------------------------------------------------------------------

// The variable min_score[i] represents the lowest score among player i and its neighbors.

// The variable deg[i] indicates the degree of player i.

// The link connection/disconnection is controlled by the parameter p.

// The variable pair represents a neighboring player j of player i while the variable pair2 indicates a neighboring player of player j.

// The variable rn1 is a uniformly distributed random number satisfying 0<rn1<1.

//--------------------------------------------------------------------------------------------------------------

if(0 < t){

for(i=0;i<m;i++){

for(j=0;j<m;j++){

pre_edge[i][j]=edge[i][j];

}

}

for(i=0;i<m;i++){ // 0:

if(deg[i]<m-1 && deg[i]>0 && min_score[i]==score[i]){// 1:

if(rn1<p){ // A:

pair=i;

while(pair==i || edge[i][pair]==0){

//=========================================

//a player is randomly selected and stored in the variable pair.

//=========================================

}

pair2=pair;

if(score[pair]<max_score[pair]){

while(1){

//=========================================

//a player is randomly selected and stored in the variable pair2.

//=========================================

if(edge[pair][pair2]==1 && score[pair2]==max_score[pair]){

break;

}

}

if(edge[i][pair2]==0 && i!=pair2){

pre_edge[i][pair2]=1;

pre_edge[pair2][i]=1;

}

}

} // end A

else{ // B:

pair=i;

while(1){

//=========================================

//a player is randomly selected and stored in the variable pair.

//=========================================

if(edge[i][pair]==1 && max_score[i]==score[pair] && i!=pair){

break;

}

}

pre_edge[i][pair]=0;

pre_edge[pair][i]=0;

} // end B

} // end 1

else if(deg[i]==0){ //2:

pair=i;

while(pair==i || edge[i][pair]==1){

//=========================================

//a player is randomly selected and stored in the variable pair.

//=========================================

}

pre_edge[i][pair]=1;

pre_edge[pair][i]=1;

} // end 2

} // end 0

for(i=0;i<m;i++){

for(j=0;j<m;j++){

edge[i][j]=pre_edge[i][j];

}

}

}
